# Helping people with psychosis to expand their social networks: the stakeholders’ views

**DOI:** 10.1186/s12888-020-2445-4

**Published:** 2020-01-29

**Authors:** Helena Tee, Stefan Priebe, Carlos Santos, Penny Xanthopoulou, Martin Webber, Domenico Giacco

**Affiliations:** 1 Unit for Social and Community Psychiatry, (WHO Collaborating Centre for Mental Health Services Development), Queen Mary University of London, Glen Road, London, E13 8SP UK; 2 East London NHS Foundation Trust, Glen Road, London, E13 8SP UK; 3 College of Medicine and Health, University of Exeter. St Luke’s Campus, Exeter, EX2 4TH UK; 4 Department of Social Policy and Social Work, University of York, Heslington, York, YO10 5DD UK; 5 Department of Health Sciences, Warwick Medical School, University of Warwick, Coventry, CV4 7HL UK

**Keywords:** Psychosis, Psychotic disorders, Social contacts, Social isolation, Social network

## Abstract

**Background:**

People with psychosis experience more social isolation than any other diagnostic group and have smaller social networks than the general population. This isolation can have a detrimental effect on quality of life. No direct, standardised interventions have been developed to specifically target this issue. Stakeholders input appears crucial in the process of developing such an intervention. This study aimed to identify the main considerations when developing an intervention aiming to reduce social isolation in people with psychosis.

**Methods:**

Focus groups and individual interviews were conducted with patients, carers and mental health staff. Data was thematically analysed.

**Results:**

Thirty four patients with psychosis, 26 carers of people experiencing psychosis and 22 mental health professionals participated in the study. Suggested aspects to be considered in a novel intervention were: i) finding and training the right staff member; ii) discussing negative social attitudes and patients’ previous negative experiences, iii) addressing personal ambivalence; iv) establishing how best to provide information about social activities; v) facilitating access to social activities, vi) striking a balance between support and independence.

**Conclusion:**

The suggestions identified can help to develop more targeted approaches to reduce social isolation within this patient group. A patient-centred approach and generic communication skills appear to be underpinning most of the helpful elements identified, whilst specific techniques and skills can help to overcome negative past experiences and motivational barriers.

## Background

People with psychosis have smaller social networks than the general population – their networks are usually composed of family members rather than friends or other contacts [4, 10]. A recent study involving 1396 patients with psychosis across 4 international sites found that 45% of those patients had not met any friend in the previous week; furthermore 35% indicated they did not have anyone they would regard as a close friend [4]. In a further study 80% of patients with psychosis reported moderate levels of loneliness and were more likely to see fewer than 2 people outside their home in a week [5].

Among the general population, people with smaller social networks have more negative health outcomes and poorer quality of life [6, 11]. For people experiencing psychosis, there is also a link with worsening symptoms [2, 4], increased hospitalisations [9], recurrence of symptoms or relapse [7] and less engagement with mental health services [12]. Additionally, people with psychosis cite social support as an essential component in the process of recovery [19].

A systematic review on interventions to improve social networks in people with psychosis demonstrated promising effectiveness for those which target social network size directly [1]. These approaches included peer support, volunteer schemes and supported engagement in an activity. Though, these schemes are often not standardised and not routinely offered across all services, making it difficult to accurately assess their impact.

In order to be effective in improving social networks, an approach needs to be simple [18], involve a mental health professional and focus on contacts outside the close family or mental health services [1]. In Italy, Terzian [16] trialled such an approach: mental health professionals supported patients to identify and engage in a social activity. The results were promising; almost 40% of participants showed an increase in social contacts, suggesting that social networks can be improved with a relatively simple, direct intervention. However, little is currently known about how such an intervention would fair in other settings and what the key components for implementation would be.

While there are promising studies on strategies that help people to identify and engage with a social activity, we do not currently have direct, evidence-based interventions to reduce isolation for this patient group. Research has shown that incorporating the perspective of patients in the design of interventions can improve their usability [15], therefore in order to standardise and design in detail an intervention which will meet patients’ needs, stakeholders’ input is crucial.

In the present study we aimed to understand the views of patients, carers and mental health professionals on what would be important to consider when developing an intervention to reduce social isolation in people with psychosis.

## Method

This study was given ethical approval by the East of England – Cambridgeshire and Hertfordshire Research Ethic Committee (17/EE/0276).

### Study design

This study consisted of focus groups and interviews where data was thematically analysed [3]. Data collection took place across three UK NHS trusts; East London NHS Foundation Trust, Devon Partnership NHS Trust and Tees, Esk and Wear Valleys NHS Foundation Trust; ensuring representation of mental health services in urban and rural areas. Separate focus groups were held at each site for patients, carers and clinicians to ensure that members could express their views without concerns related to interactions with the other groups. Individual patient interviews were also undertaken to provide wider opportunity for those patients who may not be comfortable sharing their views in a group. The study aimed to hold 9 focus groups (3 per site) and 9 interviews (3 per site).

### Recruitment

Purposive sampling [14] was used for both patients and clinicians as we wanted to ensure the presence in our sample of people with lived experience of psychosis currently under the care of secondary mental health services and those providing mental health care in the community. Convenience sampling was used to recruit carers based on self-referral to the study.

Patients were deemed eligible if they: had a diagnosis of a psychotic disorder (ICD-10 F20–29), were receiving care from secondary mental health services, could communicate in English, were aged between 18 and 65 and had the capacity to consent to taking part in research.

Carers were deemed eligible if they: were currently caring for a family member or friend who was receiving support from secondary mental health services and who had a diagnosis of a psychotic disorder (ICD-10 F20–29), had the capacity to provide informed consent, had the ability to communicate in English and were aged 18 or over.

Clinicians were deemed eligible if they: were a mental health professional with experience of providing community mental health care, were employed by a participating NHS trust, were aged 18–65, had the capacity to provide informed consent and the ability to communicate in English.

Eligible patients were identified and approached in person by members of their clinical team. Those who provided assent were contacted by a researcher and provided with further information about the study. Capacity to consent was assessed at multiple time points; it was established first by the clinician who made first contact then again at subsequent meetings with researchers, all of who were provided with appropriate training. Any concerns regarding capacity were discussed with the clinical team; no participants were excluded on this basis.

Clinicians known to the research team were approached via email or face-to-face regarding the project. Those who expressed an interest in taking part met with a researcher to discuss further.

Advertisements for carers were placed in outpatient clinics across the participating trusts and presentations were made at local service-user and carer groups. Anyone wishing to take part was invited to contact the research team so that details of the project could be discussed and eligibility established.

Prior to the study, researchers had no relationship with the participants with the exception of those in the clinicians’ focus group, some of whom were known to the research team.

All participants were asked to provide written informed consent following discussion with a researcher.

### Procedures

Focus groups and interviews took place in quiet rooms on NHS premises at the participating sites; some individual interviews took place at the participants’ home. The structure was consistent across the different focus groups and interviews. A brief description of a potential intervention was read out at the start and followed with discussion led by a topic guide. The intervention description was read by a Service User and Carer Group Advising on Research (SUGAR) who were asked to provide input on the clarity and wording of the description as well as their views on whether proposing such an intervention could have benefit, what could be improved and what possible barriers could be. The subsequent conversation informed the development of the topic guide which included questions on the content of a potential psycho-social intervention, possible barriers, suggested improvements and ways of facilitating engagement.

Focus groups were moderated by two researchers and interviews carried out by one researcher; these were male and female research assistants/associates employed at the participating sites and appropriately trained (including authors HT, PX and DG). Each focus group aimed to include 6–10 participants and last up to 90 min. Individual patient interviews lasted no longer than 90 min.

### Analysis

Focus groups and interviews were audio recorded and transcribed verbatim, with any potentially identifiable information removed. Thematic analysis, as described by Braun and Clarke [3], was conducted using NVivo qualitative analysis software to aid with coding and organisation of data.

The analysis was conducted by two researchers (HT and CS), plus a third researcher to aid with discussion and the resolution of disagreements (DG). HT has a background in psychology with a specific interest in psycho-social interventions. CS works as a community psychiatric nurse and quality improvement lead, focusing on improving service user involvement. DG is an academic and clinical psychiatrist whose research interests include social networks and developing interventions for people with severe mental illness. The multidisciplinary nature of the research team along with a rigorous analytical approach helped to ensure that interpretation of the findings remained grounded within the data.

Analysis began with an initial coding of 10% of the transcripts by HT and CS. This involved familiarisation of a sub-dataset through reading the transcripts and making note of any emerging concepts. Items with similar characteristics were then grouped together and given a label to describe the content. These descriptive labels were called “codes”. Similar codes were then grouped into “sub-themes”. At this early stage the proposed sub-themes were presented to a Lived Experience Advisory Panel (LEAP) who were asked to provide input on any items that did not make sense or should be re-worded, which items seemed most important, any items that seemed to be missing and any patterns/emerging themes.

HT and CS met to compare their codes; aiming for less than 5% discrepancy. Any disagreements were discussed iteratively, involving third researcher DG, to reach a consensus. If 95% consensus could not be reached, the same segment of transcript was read again and re-coded. This process was repeated until the discrepancy ratio was established.

Once consensus was reached, the agreed list of codes was used to form a coding framework to analyse the rest of the data. The remainder of the transcripts were coded individually by HT and CS with regular discussion between the two to ensure that no sub-theme was over or under-represented. If new codes emerged during this process, a new sub-theme was created and discussed. Once the whole dataset had been coded, an additional meeting of HT, CS and DG took place to decide whether saturation of sub-themes had been reached. At this meeting the sub-themes were grouped together to form the main themes. HT then reviewed the data included in each theme to look for divergence and areas of contestation between participant groups [20].

## Results

### Participants

Eighty two stakeholders participated in 12 focus groups and 9 individual patient interviews that were held between September 2017 and March 2018 across the three NHS trusts. This resulted in 424 pages of transcript (font size 10, single line spaced). Attendance to the focus groups ranged from 3 to 10; if attendance to a particular group fell below 5 then a second group was held to ensure that each of the participant groups were equally represented. This meant an extra focus group was added in Devon for both patients and carers and in London for staff. In total, four focus groups were held for patients, four for carers and four for staff.

Participant characteristics of the patients (from both the interviews and focus groups), carers and staff are summarised in Table 1.

**Table 1:** Participant characteristics

Interviews/Focus group: Patients (*N* = 25)	
Female gender, n(%)	44
Ethnicity, n(%)	
White British	69
Asian/Asian British – Bangladeshi	9
Black/Black British – African	9
White Other	6
Mixed – White and Black Caribbean	3
White Irish	3
Marital status, n(%)	
Single	81
Married	13
Civil Partnership	3
Divorced	3
Living situation, n(%)	
Living alone	47
Living with partner or family	28
Living with friend(s)	3
Living in shared accommodation	22
Has seen a friend in the past week, n(%)	77
Has someone they consider a close friend, n(%)	73
Focus group: Carers (*N* = 26)	
Age, mean years (s.d.)	63 (8.3)
Female gender, n(%)	77
Years spent in caring role, mean (s.d)	19 (12.7)
Relationship to patient, n(%)	
Mother/father	73
Spouse/partner	15
Son/daughter	4
Sibling	4
Friend	4
Focus group: Staff (*N* = 22)	
Age, mean years (s.d.)	44 (10.0)
Female gender, n(%)	59
Years working in mental health, mean (s.d.)	15 (9.5)
Profession, n(%)	
Nursing	27
Psychiatry	27
Psychology	5
Occupational Therapy	18
Support work	9
Management	14

The themes arising from the qualitative analysis are summarised in Table 2. The majority of themes were present in all three participant groups. Eventual intragroup saliences are specified in the description of the theme. Themes were reflected in both focus groups and individual interviews, with no specific themes arising in either of them.

**Table 2:** Themes and sub-themes

Theme	Subthemes
Finding and training the right staff member	Staff member with the right skills and attributes Should be able to work with resistance Should encourage client-practitioner rapport Should build confidence Should improve social skills Support for staff
Discussing negative social attitudes and a patients’ previous negative experiences	Negative experience with services Negative experiences with socialising Stigma Limited sense of community
Addressing personal ambivalence	Not wanting more social contacts Symptoms/nature of condition Lack of motivation Functional avoidance Lack of social skills Socialising as generally difficult
Establishing how best to provide information about social activities	Accessible information Discussions around the information provided Manageable activities Learning from past experience Builds on patient interests
Facilitating access to social activities	Cost Travel Physical disabilities Lack of available activities Access to information Language
Striking a balance between support and independence	Supporting with pragmatics Encourage independence Providing the right kind of support Involve carers Personalised

### Finding and training the right staff member

The importance of finding the right staff member to work with patients on improving their social networks, in terms of their role and attributes, was discussed widely. A common focus was the skills that the staff member should have such as interpersonal and communication skills, or the necessary attributes such as patience and empathy.

Participants also spoke of the role such a staff member would have within mental health services. Some suggested that this person should already be involved in a patient’s routine care.

“You can’t just let a stranger into the circle because it’s [about] trust. Do we trust that person?” – London Carers

Whereas some felt that this person should have a separate and specifically defined role.

“…if it’s someone that the client already works with, then they’re going (…) to talk about all those other things that they normally do…” – London Patient (I)

Many spoke to the importance of the staff member being able to identify and work with potential barriers expressed by the patient. This should be handled by getting to know a patient and understanding their reasons for what could be perceived as a lack of engagement.

“The clinician or the person (…) needs to be aware of the problems; access, transport, toileting. They need to be aware of disablement in this particular area.” – York Carers

The relationship between the staff member and patient was seen as particularly important for facilitation. Encouraging rapport was seen as a way of creating positive experiences with services, which would in turn increase the effectiveness of an intervention.

“Because the caseworker will have got to know them, (…) it’s the relationship which is the whole thing, it’s massively important.” - York Carers

Staff should also be able to work with patients on building their confidence and improving their social skills.

“…so that they have the communication skills, understand about listening, about being part of a group (…) and then also handling and coping with sometimes people are rude. (…) I think really that’s the whole point (…) to build up people’s own skills.” – London Patient (I)

Training for staff was considered essential. It was also highlighted how challenging this role could be and how staff should be supported through regular supervision.

“I definitely think as the person delivering it you’d need to (…) have almost peer supervision where you can discuss what was helping, what was going badly and what was going well” – Devon Staff

### Discussing negative social attitudes and patients’ previous negative experiences

A recurrent theme was how negative experiences with mental health services can prevent patients from wanting to engage further. This can be due to negative relationships with the care team, a perceived loss of support due to funding cuts and staffing issues, or a dislike of their care being ‘medicalised’.

“My experience with mental health care professionals is they can be fairly judgemental, fairly controlling…” – London Patient (I)

“…you want people to treat you as a whole person, not your illness -- so as soon as you’re interacting with someone who’s titled as a mental health professional we’re talking about your diagnosis, we’re not talking about you as a person.” – Devon Carers

Staff and patient expectations regarding socialising and engaging in activities was a common conflicting issue. Staff may encourage patients to socialise more than they actually want to, leading to staff frustration and perceiving patients as “difficult to work with” or “not engaging”.

“What comes to my mind is the frustration (…) that they are not progressing as quickly as you would want them or, they are not engaging as you would want them and actually it helped to distinguish -- what’s your wish for them?” – London Staff

Participants across all focus groups discussed how negative experiences in social situations can lead patients feeling reluctant to engage in further activities.

“…they’ve been harassed, (…) they’ve been tortured in many ways through their experiences with other people (…) that is going to have some effect on (…) what they would want to participate in.” – York Staff

A common theme was about how society may discourage some patients from wanting to socialise. This can be due to the perceived stigma associated with mental illness.

“…they might think like “oh what if I go and try this activity, what if someone finds out that I’ve got mental illness?” And they might find it weird or be alienated about that.” – London Patient (I)

Others discussed how society displays a limited sense of community, making it difficult for patients to feel integrated.

“All these people, they’re all individuals, they’re all, they live in different places. They act on their own. They’re not part of a community.” – Devon Carers

### Addressing personal ambivalence

Patients may show ambivalence about whether they want to increase their social networks, any intervention should help to identify and acknowledge this ambivalence. Some participants in the patient focus groups and interviews stated that they had no desire to have more social interactions and that others may feel the same, either because their current network was sufficient, or because they preferred their own company. Some felt that this ambivalence could arise from a number of factors which may be related to the experience of psychosis, particularly when someone is acutely unwell. Factors include fear of going out, low self-esteem and concerns about getting overwhelmed.

“When I was really ill I wouldn’t talk to anyone, I wouldn’t feel like talking or meeting people.” – Devon Patients (FG)

Ambivalence can present as a lack of motivation, which was seen as difficult for both staff and patient to manage. However, participants noted that resistance is usually functional and often used by patients as a way of keeping themselves safe.

“Because I’m fearful about leaving the house…I take a calculated risk going out on random days with key workers for a short period of time.” – Devon Patient (I)

It was also noted that socialising can be a challenge, even for those not experiencing mental illness.

“…everyone gets anxious when you go and do something new and meet new people. That’s normal….” – Devon Staff

### Establishing how best to provide information about social activities

The way information on social activities is provided was a dominant theme. It was emphasised that information should be accessible; which may involve clearly written information or having a discussion around what is provided. It was felt that information should focus on activities that are manageable and meaningful to the patient.

“I think it very much depends on the individual preferences of the person… probably activities that (…) are meaningful to the person…” – London Patient (I)

When considering potential activities, learning from patients’ past experience can be helpful. This could involve enquiring about what activities a person has already engaged in, emphasising that decisions should be led by patient interest.

“I suppose the discussion could include people talking about their life before they became unwell (…) talking about what aspects of their life they particularly want to recover and how social contact and activities can promote that.” – London Patient (I)

### Facilitating access to social activities

Accessibility was a consistent theme; both in terms of information and the activities themselves. A common concern was patients struggling to access activities because of cost, difficulty travelling and physical disabilities, though concerns about disability were more prevalent from the carers.

“It’s all right giving them a list of things to do and taking them to an activity, but if they don’t have the money to pay for it they won’t do it.” York Patient (I)

“There aren’t the facilities or activities that can cater for the disabled person with psychosis.” – York Carers

Some also feared that there was a lack of social activities consistently available, and information about how to access those activities can be limited. These concerns were particularly salient for the more rural sites.

“Because when one of you just said oh there’s lots of things that exist, well maybe they do but my daughter doesn’t know what they are, and I don’t know what they are.” – York Carers

Participants were also concerned about language barriers potentially exacerbating social isolation. This was most salient in the staff focus groups in London, potentially reflecting the diverse populations that staff in this area work with.

“With communities that do speak their language, it could be (…) that people have relied on a family member to take them out and about and that person died (…) suddenly they are left on their own.” - London Staff

### Striking a balance between support and independence

Providing the right level of support was a salient theme across all groups, often with differing ideas about what constitutes the right amount, especially when balancing support with encouraging independence. One view emphasised that patients would need ongoing support from staff in order to be able to attend social activities. This was referenced both in terms of the pragmatics of attending an activity (e.g. how to get there) and providing encouragement. Suggestions for providing further support included longer and more frequent meetings with staff, additional telephone follow-ups and reminding patients about upcoming activities.

“I think with some patients. I think they tend to contact their care coordinators regularly (…) because they might not feel comfortable with their personal life and they’re going to be like “I need help with this”. – London Patient (I)

“We’re starting almost from a premise of trying to make people independent, and I don’t think people with mental ill health are best with that as a focus. I think being interdependent in relationships with people is key to mental wellness” – York Carers

An alternate view stressed the importance of not allowing support to lead to a dependence on staff and that encouraging patients to try and do things themselves should be a priority.

“It’s a reservation I have about the whole supporting people into activities idea, (…) I think it can possibly reduce people’s incentives to find out for themselves where some activity might be and also I think it might reduce their incentive to create their own social circle.” – London Patient (I)

Finding the balance between dependence and independence seemed crucial. It was felt that this balance was important in giving patients “a little push” to step out of their comfort zone without overstretching them.

“Some people want to be pushed, some people don’t. I would lock myself indoors because of the paranoia or whatever, and the voices, I didn’t want to go out. I didn’t want to socialise (…) now I’m a bit more open minded to learn new things and do the things that I once enjoyed.” London Patient (I)

The capacity to reach certain groups of patients, namely those who may not be acutely unwell but still require support was felt to be important. This group was often seen as falling through the gaps of current services whereas they could be targeted through the implementation of specific interventions.

“There are a lot of people, (…) who are not symptomatic, they are just isolated. They are taking their medication and by default these are the people who are not making much fuss (…) they’ve slipped through the net.” – London Staff

It was felt that keeping the intervention direct and focused would allow people to engage. It was also seen as helpful for the intervention to have a structure, as the sense of routine could be beneficial.

“It’s good in a way to keep it short because hopefully it will really focus people (…) and concentrate them.” – London Patient (I)

Providing the right amount of support could also be facilitated by embedding the intervention in to current services and aligning with their existing goals, this was largely talked about by staff who reflected on the state of current services.

“I think there is definitely a network of people (…) that could really help and the infrastructure is already there. It wouldn’t need too many extra resources.” – London Staff

It was suggested that support from carers should also be utilised, as involving them could help to facilitate engagement. This subtheme was discussed solely in the carers’ focus groups.

“I think all this needs to include the family very much.” – York Carers

There was no consensus on how much support should be provided. Instead, a commonly held view was that an intervention should be personalised to consider individual needs. This was seen as particularly important as patients may vary in terms of how much support they need as well as what level of social interaction they are aiming for.

“It's horses for courses, because everyone is ill in a different way and that, so, it depends how you feel as a person and what you need.” – Devon Patients (FG)

“I think it needs to be a person centred approach, so you need to know what the individual will benefit from. Some people, like you said, might just want to have that stability, knowing exactly the routine. Other people perhaps need to come out of that routine in order to expand their horizon and do different things.” – London Carers

## Discussion

This study was able to identify key factors that need to be considered when developing an intervention to increase social networks in people with psychosis. The findings support those of previous studies [1, 16] which suggest that a direct and focused approach can be helpful in tackling this issue. The themes identified suggest future directions for development and implementation of helpful clinical interventions.

While views of stakeholders were sometimes conflicted across different focus groups, a general consensus was the importance of a patient-centred approach as reflected in the suggested need to strike a balance between support and independence, the need to provide tailored information and the need to improve accessibility. A potentially acceptable and helpful approach should allow the patient to lead the conversation, adjusting the level and type of support provided where necessary.

While various suggestions were made about what the job role of the staff member most appropriate to deliver an intervention would be, the traits and skills they should have were generally agreed on. Having a mental health professional that was empathetic with good communication skills was seen as crucial by everyone, more so than what specific job role they should have. Having experience of dealing with some of the challenges presented was also seen as an important factor to address social isolation with this group, particularly considering the resistance they may face and the challenge of navigating this with patience and understanding. This suggests that, with appropriate support and training, an intervention supporting patients with psychosis to expand their social networks could be delivered by a range of mental health professionals.

Negative personal experiences which can arise from feeling they have been treated badly by those in society or those working in services may generate ambivalence towards wanting to socialise or engage in service-led interventions. These experiences should be better acknowledged and understood by any staff members who are working with patients to improve their social networks, incorporating the views of patients on what would work best for them would help to keep the discussion person-centred. In this regard, resource-oriented approaches, such as Solution Focused Therapy, could be a helpful way of utilising a person’s own strengths and expertise to move towards solutions to these problems [13]. Furthermore, the addition of motivational interviewing could address potential ambivalence and strengthen motivation for change [17]. These two approaches share a number of commonalties: both endeavour to reframe resistance, both focus on strengths and positive change, and both can be incorporated in to time-limited interventions [8]. When used in conjunction, Solution Focused Therapy and Motivational Interviewing could be effective in changing behaviour even in the presence of lack of motivation and ambivalence.

Accessible information was seen as crucial, while opinions on the format of information varied. What could be agreed on was that informing patients of activities that are manageable and of relevance to them would be helpful; there would need to be an understanding of any practical barriers that may inhibit access to the activity. This could be done through structured information provision, allowing patients to explore their interests and take the lead in what options for activities should be explored. Informative materials could then be provided to best meet the needs of the individual.

### Strengths and limitations

To our knowledge, this is the first study aiming to collect the views of stakeholders on developing ways to reduce social isolation in people with psychosis. The strengths of this study are the inclusion of a range of participant groups, a flexible methodology (both interviews and focus groups) and the reporting of results according to the consolidated criteria for reporting qualitative research (COREQ). Efforts were made to gain insights from all groups (patients, carers and staff) who would likely have a role in this type of intervention across both rural and urban areas. Focus groups allowed us to make use of interactions between participants in generating ideas, while offering 1:1 interviews meant that the views of those who may struggle with social interactions could be heard. Potential bias arising from the background of the researchers was minimised by seeking advice from a Lived Experience Advisory Panel (LEAP) during the analysis, though including a researcher with lived-experience at the data collection stage could have been helpful in eliciting divergent opinions within and between the groups.

Limitations include the high likelihood of selection bias; those more open to participating in service-led interventions may have been more willing to take part in the focus groups, especially considering they were held on trust premises by staff affiliated with services (though individual patients were offered the option of being interviewed at home). Furthermore, those patients who are most socially isolated were more difficult to approach as they may less likely to attend appointments. Similarly, staff who took part may see social isolation as a more salient issue and be more in favour of developing interventions, meaning that those with more negative views of such a strategy could be under-represented. This potentially contributed to the consensus amongst participant groups of the main considerations; including participants with a less favourable view on the topic could have resulted in more divergence. Furthermore, all authors have a background in mental health practice or research and our focus was on interventions delivered by health services. It is possible that this focus may have precluded the exploration of other avenues for expanding social networks of people with psychosis. We strongly suggest that interventions which are delivered outside of health services or are explored in future conduct research in this area. The discussion was also largely theoretical; participants were provided with a description of a potential intervention but would not have experience of actually participating. However important the theoretical background work may be, the novelty of the therapeutic approach suggested may mean that the concept is unfamiliar to many; hence the considerations will need to be substantiated by actual feasibility testing of interventions in real practice.

## Conclusion

Stakeholder engagement through focus groups and individual interviews has provided relevant insights on how to help people with psychosis expand their social networks. A patient-centred approach appears to be a core principle, whilst more specific therapeutic techniques may be required to overcome ambivalence and the negative effects of previous experiences. Practical barriers should not be underestimated and the provision of appropriate, user-friendly information, as well as an understanding of access-related barriers, should be considered in order to maximise the chances of success of an intervention to improve social networks in this population.

## Data Availability

The datasets used and/or analysed during the current study are available from the corresponding author on reasonable request.

## Abbreviations

LEAP: Lived Experience Advisory Panel
SUGAR: Service User and Carer Group Advising on Research
